# Interactive wiimote gaze stabilization exercise training system for patients with vestibular hypofunction

**DOI:** 10.1186/1743-0003-9-77

**Published:** 2012-10-09

**Authors:** Po-Yin Chen, Wan-Ling Hsieh, Shun-Hwa Wei, Chung-Lan Kao

**Affiliations:** 1 Department of Physical Medicine & Rehabilitation, Taipei Veterans General Hospital, 201 Shih-Pai Road, Section 2, Taipei, 11217, Taiwan; 2 Center for Geriatrics and Gerontology, Taipei Veterans General Hospital, 201 Shih-Pai Road, Section 2, Taipei, 11217, Taiwan; 3 Institute of Physical Therapy and Assistive Technology, National Yang-Ming University, School of Biomedical Science and Engineering, No. 155, Sec 2, Linong Street, Taipei, 11221, Taiwan; 4 School of Medicine, National Yang-Ming University, No. 155, Section 2, Linong Street, Taipei, 11221, Taiwan

## Abstract

**Background:**

Peripheral vestibular hypofunction is a major cause of dizziness. When complicated with postural imbalance, this condition can lead to an increased incidence of falls. In traditional clinical practice, gaze stabilization exercise is commonly used to rehabilitate patients. In this study, we established a computer-aided vestibular rehabilitation system by coupling infrared LEDs to an infrared receiver. This system enabled the subjects’ head-turning actions to be quantified, and the training was performed using vestibular exercise combined with computer games and interactive video games that simulate daily life activities.

**Methods:**

Three unilateral and one bilateral vestibular hypofunction patients volunteered to participate in this study. The participants received 30 minutes of computer-aided vestibular rehabilitation training 2 days per week for 6 weeks. Pre-training and post-training assessments were completed, and a follow-up assessment was completed 1 month after the end of the training period.

**Results:**

After 6 weeks of training, significant improvements in balance and dynamic visual acuity (DVA) were observed in the four participants. Self-reports of dizziness, anxiety and depressed mood all decreased significantly. Significant improvements in self-confidence and physical performance were also observed. The effectiveness of this training was maintained for at least 1 month after the end of the training period.

**Conclusion:**

Real-time monitoring of training performance can be achieved using this rehabilitation platform. Patients demonstrated a reduction in dizziness symptoms after 6 weeks of training with this short-term interactive game approach. This treatment paradigm also improved the patients’ balance function. This system could provide a convenient, safe and affordable treatment option for clinical practitioners.

## Introduction

Dizziness is a complex feeling that is often characterized by a combination of poor spatial orientation and a sense of unsteadiness. Dizziness is frequently accompanied by postural imbalance, which can result in an increased risk of falls [1,2]. Previous studies have revealed that nearly half of the adult population with dizziness over the age of 40 could suffer from vestibular system problems [3]. Peripheral vestibular hypofunction is one of the major causes of dizziness. Impaired function of the vestibular system can cause retinal slip and decreased dynamic visual acuity (DVA), which can lead to gaze instability and blurred vision during head rotation. The treatment of patients with dizziness remains a major challenge in clinical practice.

Among the many vestibular system reflexes, the role of the vestibulo-ocular reflex (VOR) is to produce eye movements in the direction opposite to that of head rotation to stabilize the image in the retina, avoid retinal slip and maintain visual acuity. Visual acuity is reduced by retinal slips exceeding 2 degrees per second (^o^/s) [3-6]. When the speed of the head rotation exceeds 100^o^/s, only the VOR can adapt quickly enough to stabilize vision [7,8]. Therefore, under certain circumstances, such as looking around while walking or walking in complex dynamic environments (e.g., a crowded shopping mall or a metro escalator), patients with peripheral vestibular hypofunction will experience dizziness, which often significantly impacts their quality of life. A previous study by Herdman [9] examined vestibular adaptation after repetitive head movements in patients with unilateral and bilateral dysfunction. Herdman’s study showed that vestibular exercise is the only effective method to improve DVA. The function of gaze stabilization training, which is one of the vestibular exercises that have been developed, is to improve visual acuity through the increase in VOR gain (eye velocity/head velocity). Traditional methods of gaze stabilization exercise include the following: 1) maintain a stable gaze on a dynamic or stationary target during up-down or side-to-side head rotations; and 2) perform alternate rotation between two targets at a distance, whereby the eyes look at the target first, followed by a head movement to the same target. The goal of the gaze stabilization exercise is to improve the interaction between the visual and the vestibular systems during high-velocity head movements. Although gaze stabilization is a simple exercise that can be performed at home, the vestibular effect cannot be achieved unless the head movement velocity reaches 120-180^o^/s, which is difficult for patients to estimate while exercising. Therefore, specialized sensory equipment is typically required to assist with these training exercises.

With the recent developments and improvements in sensory devices, wireless communication technology and three-dimensional computer simulation technology, new therapeutic methods involving the application of human-computer interface strategies have been developed. For example, virtual reality (VR) therapy has been gradually developed for use in medical rehabilitation therapy, including therapy for stroke and spinal cord injury patients [10-12]. Viirre [13,14] and Kramer et al. [15] were the first to investigate the application of VR therapy in patients with vestibular dysfunction. The VR technique provides patients with a controlled environment to help them gradually adapt to situations that typically induce symptoms [16]. This technique has been applied in a limited number of patients with visual vertigo and has been proven to be effective [17]. The Nintendo Wii® is an affordable computer gaming system that has gained widespread popularity. The Wii system integrates a user-centered design concept and has drawn considerable attention from the field of medical rehabilitation [18]. Previous studies have used the Nintendo Wii balance board (WBB) to train and assess balance in elderly patients [19,20]. The WBB in combination with VR rehabilitation has also been applied in the treatment of patients with brain injuries and stroke. The results from these studies indicate that patients exhibit significant improvements in static and dynamic balance and life function [21,22]. To establish a more convenient exercise training system, the objectives of this study are to quantify head movements in patients with peripheral vestibular dysfunction and to create training stimuli with varying degrees of intensity and difficulty using controlled game parameter combinations. Therefore, we established a vestibular function rehabilitation system by coupling infrared LEDs to an infrared receiver of a Nintendo Wii. We compared the perceived dizziness level, DVA and balance function parameters of patients before and after 12 sessions of exercise training. We followed up with patients 1 month after treatment to further investigate the continuous efficacy of rehabilitation.

## Methods

### Participants

Three unilateral and 1 bilateral vestibular hypofunction patients participated in this study. The diagnosis of vestibular hypofunction was based on the results of a head thrust test, horizontal head shaking nystagmus test and caloric test (AIRSTAR, Micromedical Technologies, Illinois, USA). All participants were asked to sign an informed consent form, and the Taipei Veterans General Hospital Institutional Review Board approved the study. Exclusion criteria included subjects with benign paroxysmal positional vertigo, post-traumatic vertigo, degenerative neurological disease, whiplash injury, neck pain and cognitive impairment. All 4 participants underwent thorough neurological examinations. None of the participants exhibited any neurological signs (motor weakness, increased deep tendon reflex or nystagmus) of central nervous system lesions. Therefore, no neuroimaging was performed.

### Clinical assessments

#### Dizziness handicap inventory (DHI)

The DHI [23] is a validated 25-item scale questionnaire, with scores between 0 and 100, used for the evaluation of problems derived from dizziness. A higher DHI score indicates a greater level of handicap. The test-rest reliability from 95 patients was 0.92 to 0.97 [24].

#### The activities-specific balance confidence scale (ABC)

The ABC scale [25] is a balance confidence scale. The average of 16 items related to functional daily activities was recorded to reflect the subjective feeling of dizziness. Test-retest reliability indicated an intra-class correlation coefficient (ICC) of 0.87 (95% CI, 0.76 - 0.93) for the Chinese versions [26].

#### The hospital anxiety and depression score (HADS)

The HADS [27] is a self-assessment, 4-point scale questionnaire with 14 items for the measurement of psychological impacts on patients. A higher score indicates a higher level of anxiety and depression. The sensitivity and specificity for both HADS-A and HADS-D are approximately 0.80 [28].

#### Visual analogue scale (VAS)

Subjects were asked to rate their dizziness severity from 0 to 10. A higher score indicates greater severity.

#### Tinetti fall risk performance scale (POMA)

This is a 2-point scale used for the evaluation of static and dynamic balance functions. Scores lower than 19 indicate a high risk of falling, and scores greater than 25 indicate a low risk of falling [29]. The concurrent validity of POMA is 0.64 - 0.70, and the inter-rater reliability was 0.75 to 0.90 [30].

#### Dynamic gait index (DGI)

The eight-item DGI is based on a 4-point scale used for the evaluation of balance in different walking situations. Scores lower than 19 indicate a high risk of falling [31,32]. The test-retest reliability was 0.86 to 1.00 for vestibular patients [33]. The concurrent validity was 0.71 between the DGI and the Berg Balance Scale [34].

#### Timed “Up and Go” test (TUG)

The total time required for subjects to stand from a chair with armrests, walk 3 meters as quickly and as safely as possible, turn around, walk back to the chair and sit down was measured. The cut-off value for the general population is 13.5 seconds [35]. For patients with vestibular hypofunction, the cut-off value is 11.1 seconds [36].

#### Sensory organization test (SOT) and dynamic visual acuity (DVA) assessments

The sensory organization test and DVA were assessed using the Smart Balance Master® (NeuroCom® International, Inc., Oregon, USA). The sensory organization scores were evaluated to assess the subjects’ ability to use sensory input for balance and to perceive objects during head movements. The static visual acuity varies from 0 to 0.1 logMAR. The DVA for the age group 20 to 29 years was 0.02 logMAR. The intra-class correlation coefficient of the DVA was 0.83 for the vestibular population [37].

### Rehabilitation instruments

Nintendo Wii® is a television game console that was officially released at the end of 2007. It includes 2 to 4 control handles (the Wii remote or Wiimote), which are connected to the console through Bluetooth wireless transmission. An important function of the Wiimote is to serve as a location tracker similar to a computer mouse, with precise pointing and aiming ability. The head of the Wiimote uses a built-in infrared CMOS camera as a sensor to capture external infrared light sources. Infrared light is emitted through the sensor bar connected to the Wii console. The sensor bar acts as a position indicator and determines the direction in which the Wiimote is pointing. The CMOS camera is a monochrome sensor with an infrared filter that can only be passed by external infrared sources with wavelengths of up to 940 nm. A built-in image processor tracks the received light as four dots, and the data sent to the console represent only the positions of these dots. In the present study, we designed an infrared-emitting cap that fixed an infrared light source on the user’s occipital bone and utilized the features of the Wiimote to detect the position of the infrared lights during the user’s head motion.

#### Wiimote connection

The readings of each sensor of the Wiimote and the signals controlling the feedback components are transmitted through a Bluetooth Human Interface Device Profile (Bluetooth HID). Bluetooth HID employs the USB HID protocol to provide an interface between an operating system and user-input devices, such as a keyboard, mouse or joystick. Hcitool can be used to scan for surrounding Bluetooth devices and display the corresponding information, including device names and Bluetooth addresses. When a Bluetooth adapter is connected to a Wiimote, the Bluetooth address of the Wiimote can be acquired using Hcitool, and signals from the Wiimote can be processed using the LabVIEW program. The computer can obtain relevant information, such as the device name of the Wiimote (Nintendo RVL-CNT-01) and the Wiimote-supporting HID protocol. The program developed in this study can receive signals from the Wiimote and perform real-time analysis of the data from the subjects’ head movements.

#### Head movement detection

**Figure 1:**
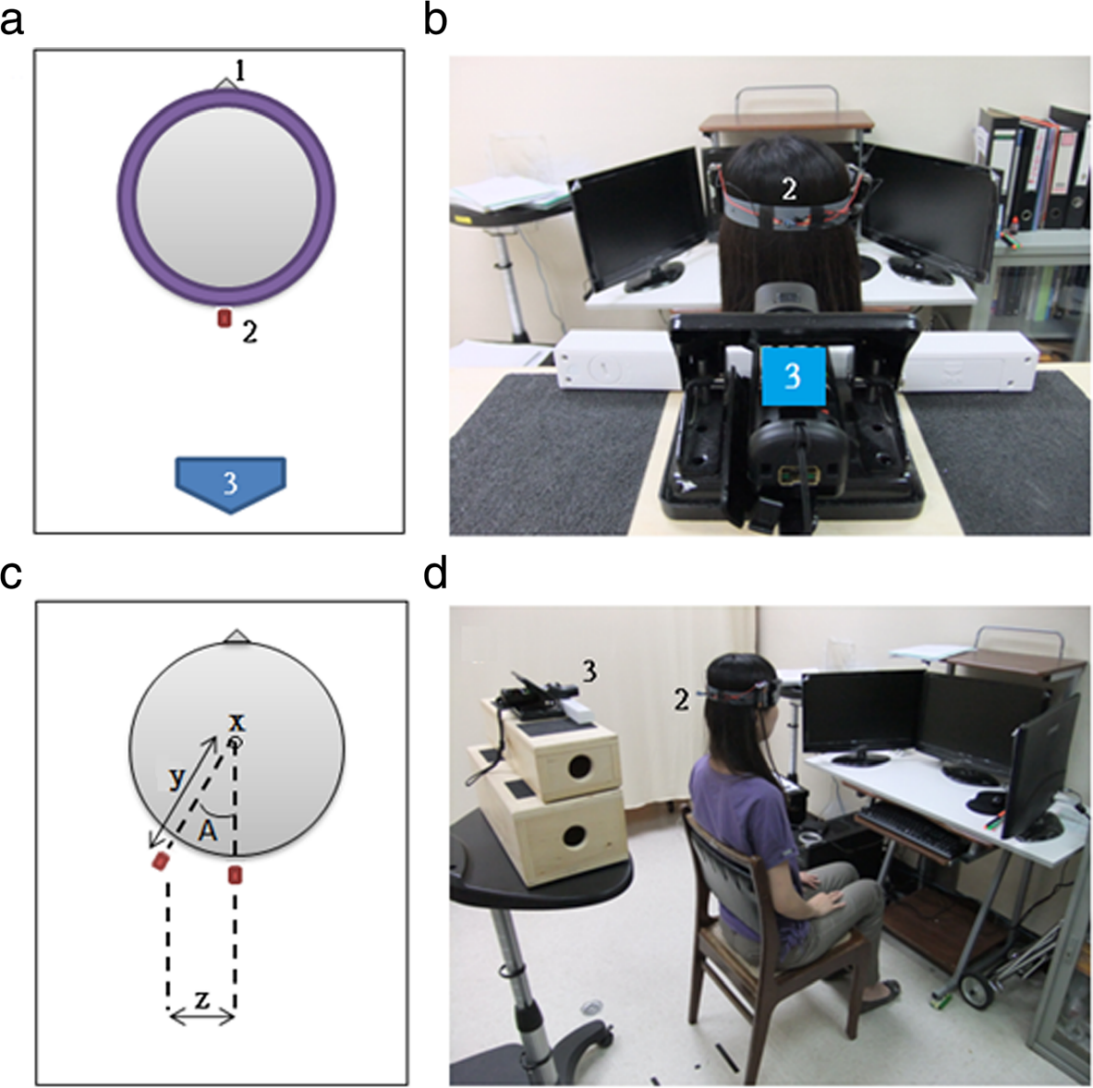
**a) A diagram of the system illustrating the following: “1” indicates the direction that the subject is facing, “2” indicates the infrared-emitting LED, and “3” indicates the infrared receiver.b**) A photo of the system shows the positions of 2 and 3. **c**) A diagram of the subject’s head rotation. The angle of the head rotation is calculated through trigonometric functions using the known length y and the measured length z. **d**) An overview from one side of the system.

As shown in Figure 1, the subject wore a cap with an infrared-emitting LED. The LED was located at the center of the back of the head, approximately 2 to 3 centimeters above the level of the eyes, and the horizontal strap was parallel to the ground. The Wiimote was placed approximately 40 centimeters away from the back of the head and was connected to the PC through Bluetooth. This system displayed the relative position and displacement of the rotated infrared LED in real time. The center of the subject’s head was used as the center of rotation by the system, and the angular speed of the synchronous head movements of the subject were calculated in real time. The program designed in this study is illustrated in Figure 1.

#### Interactive training

Using multi-screen technology, the system designed scenes customized to the user and presented the daily life of the user through videos on a three-screen system. The screens were arranged in front and to the left and right of the user to establish virtual, task-specific effects and make the training results more transferable [38,39].

**Figure 2:**
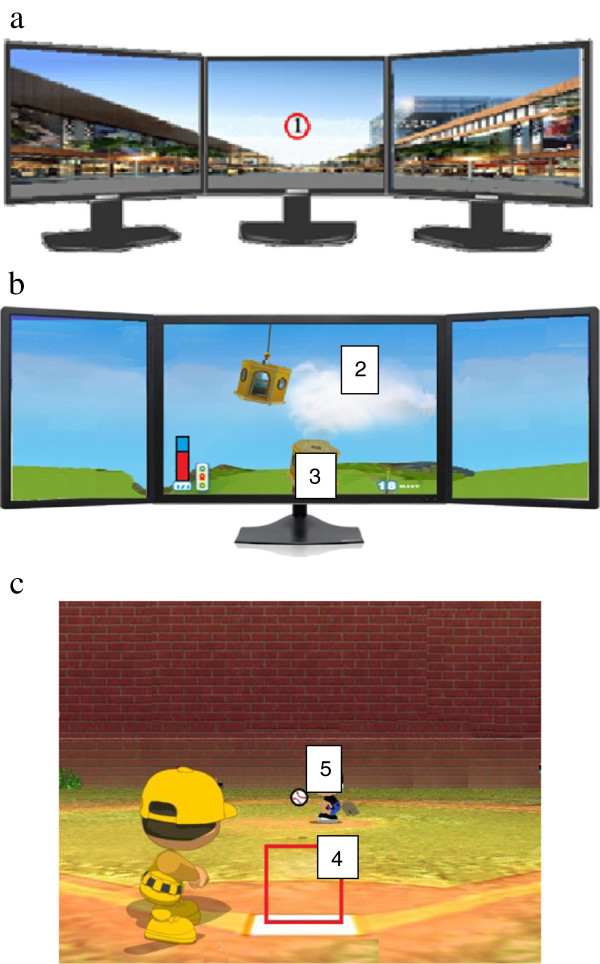
**The two training interfaces of this system were designed as follows: a) the visual stability test for the patients using street scene backgrounds with a red target circle [**[1]**] as the focal point, and b) an interactive game, in which the patient’s head-turning speed controls accurately dropping a swinging box [**[2]**], onto a target[**[3]**]. c**)The red frame [4] is the target region indicating when the baseball [5] should be hit. The subject must turn his or her head quickly when the ball enters the target position to achieve a hit.

As soon as the subject put on the detection system after the calibration of position, the computer immediately detected the head rotation speed, which was closely monitored by a professional physical therapist. Two types of training games were designed for this study. The first type consisted of common street scenes or daily-life-related images. The subject focused on the center of the screen and quickly turned his or her head left and right to perform visual stability compensatory movements. A target number (font size 20) was presented when the head-turning speed of the subject reached the speed required by the treatment. The subject had to correctly identify the number that he or she had seen or the required head-turning speed would be reduced by 10 degrees per second. Conversely, the required head-turning speed would be increased by 10 degrees per second after three consecutive correct answers. Turning the head at a speed of 120-180 degrees per second was the goal of vestibular function training (Figure 2a). The second type of training included two interactive games. In the first game, the eyes of the subject should be fixed on the swinging square. When the square moved near the target area below and the subject’s head-turning angular velocity reached the training threshold, the square immediately dropped to the target area below, where the squares would stack up (Figure 2b). If the squares were not stacked up straight, then the target area would sway, and the stacks would fall. The game task was accomplished when the stack of squares reached a certain height (10 to 20 squares). As the subject’s performance improved, the head-turning speed requirement was gradually elevated to a turning speed greater than 120 degrees per second.

In the second game, a pitcher at the top of the screen threw a baseball toward a batter, who was the target of the patients’ visual attention. The patients initially focused on the baseball in the pitcher’s hand. When the baseball flew near the front of the batter after being pitched, the patient immediately turned his or her head quickly. When the head-turning speed reached the target speed, the batter would swing the bat to hit the baseball, which was counted as the accomplishment of this single game task. The pitch timing and ball velocity would be changed randomly to increase the difficulty of training. The game was scored as the number of successful hits out of 10 attempts (Figure 2c).

A professional physical therapist controlled the game difficulties and monitored and adjusted the required patient head-turning positions and velocities throughout the entire training process.

#### Rehabilitation training

The training participants received computer-aided vestibular rehabilitation training for 2 days each week for a period of 6 weeks. Each day, training lasted a total of 30 to 40 minutes. VAS assessments were administered to the participants prior to exercise. Training began with a side-to-side head movement of approximately 60^o^ at a speed of 90^o^/s. Over the first 2 weeks, the head movement speed increased to 120^o^/s. The participants changed positions from sitting to standing and from standing on a stable floor to standing on an unstable cushion surface.

Each individual training session lasted 5-6 minutes, with a break interval of 1-2 minutes among sessions. Approximately 4-5 rounds of training were performed each time. Three games were performed alternately.

The instructions for the home program were as follows: 1. Stand firmly on a steady surface, draw a cross on a card and hold the card at eye level. Stare at the cross while turning your head from side to side without losing focus. Move your head as fast as you can for 1 minute. 2. Stand firmly on a steady surface, draw a cross on a card and hold the card at eye level. Stare at the card while moving the card from side to side, and move your head in the opposite direction. Move your head as fast as possible, and keep staring at the card as long as possible. Continue for 1 minute before taking a short break. Practice these two exercises for 30 minutes every day.

## Results

The results of all assessments (initial, post-treatment and 1 month post-treatment assessment) are shown in Table 1.

**Table 1:** Assessment results for pre-, post and one month follow up of computer-aided vestibular training

		**SOT**	**DVA**		**POMA**	**DGI**	**VAS**	**HADS**	**DHI**	**ABC**	**TUG**
			**R**	**L**							
Case1	pre	54	0.14	0.3	21	16	4	29	52	6.19	7.2
female	post	77	0.02	0.2	24	18	1	6	14	9.25	6.5
62 y/o	Follow-up	67	0.016	0.016	23	20	1	8	10	9.31	6.78
Case2	pre	53	0.48	0.5	15	13	5-7	32	54	4.44	12.58
female	post	70	0.32	0.28	24	17	3	12	24	6.81	10.58
78 y/o	Follow-up	71	0.12	0.22	24	19	2	19	12	7.38	10.32
Case3	pre	56	0.2	0.7	23	13	4	23	66	6.31	13.85
male	post	83	0.06	0.26	27	19	1	17	36	9.12	11.42
67 y/o	Follow-up	80	0.10	0.18	25	18	1	16	24	9.31	10.9
Case4	pre	81	0.5	0.6	25	18	5	26	50	9.5	9.5
female	post	83	0.1	0	28	23	1	16	24	9.69	8.23
40 y/o	Follow-up	78	0.22	0.22	28	22	2	18	28	9.6	7.96

*SOT*: Sensory organization test, *DVA*: Dynamic visual acuity, *POMA*: Tinetti Fall Risk Performance Scale, *VAS*: Visual Analogue Scale, *HADS*: The Hospital Anxiety and Depression Score, *DHI*: Dizziness Handicap Inventor, *DGI*: Dynamic Gait Index, *ABC*: The Activities-specific Balance Confidence Scale, *TUG*: Timed “Up and Go” test.

### Patient 1

#### Brief history and laboratory data from the initial assessments

This patient is a 62-year-old female who works in city government. Her main job is to maintain order and serve as the directory consultant for various exhibitions. Approximately one year before the start of this study, she experienced dizziness, ringing in the ears and swollen scalp symptoms when moving her head. In addition to these symptoms, she also felt unbalanced when walking fast. The symptoms were particularly severe when she moved her head from right to left. She experienced nausea and even vomiting when looking out the window while riding public transportation. She participated in a vestibular rehabilitation program for 3 weeks approximately 11 months prior to the start of this study. Her symptoms improved slightly, and she returned to work immediately. However, she was still unable to tolerate her workload and therefore volunteered to participate in this study. The caloric test showed 37% left-sided canal paresis on initial examination.

#### Training progress

At the beginning of the training program, she was only able to perform training while seated in a completely steady position. She started feeling nauseous and the urge to vomit whenever her head movement speed exceeded 90^o^/s after approximately 30 seconds. After the second week, she gradually became able to perform training with speeds of 100-120^o^/s while seated. She also began video game training in a standing position, and she gradually extended her training time to 3-5 minutes per training session. However, significant upper body movement was still observed. On the 4^th^ week of training, the patient was able to maintain her training performance with head movement speeds of 120^o^/s in a tandem stance. The balance board and tilt board were gradually added to interfere with her standing balance. She was able to tolerate the challenge by the second training session in the 6^th^ week and was able to maintain good balance as well.

#### Follow-up assessments

When examined 1 month after training, the patient quickly completed the DGI and POMA assessments. Although she still exhibited an aberrant distance between her feet while walking, her movement speed was clearly improved. The patient also did not experience any dizziness when looking out of windows while riding public transportation. She did not report feeling any uncomfortable symptoms, such as dizziness, when she increased her concentration while quickly turning around in a crowded environment. She felt that it was much easier to perform her job because her walking speed and stability had both improved.

### Patient 2

#### Brief history and laboratory data from the initial assessments

This patient is a 40-year-old male who works in an office and spends most of his time seated behind a desk. Approximately 2 years prior to the start of this study, he caught a bad cold, after which he began to experience severe dizziness. He then began experiencing a variety of symptoms, including vertigo, ringing in the ears, headaches, severe nausea and vomiting, especially while performing rolling or rotation movements. His symptoms were particularly serious in the evening. He felt a loss of ability to focus on his surroundings and experienced difficulty concentrating on targets. In addition, he reported feeling particularly dizzy when performing head rotations while engaged in close dialogue with others, and he felt especially uncomfortable in crowded places. His family members also noticed that he could not walk or swim in a straight line. He gradually lost the ability to perform the head movements necessary for breathing while free-style swimming. The patient emphasized that his balance was good prior to the onset of the disease and that he had not previously experienced symptoms of dizziness. His dizziness had become a major obstacle in his work and daily life. The caloric test results 1 week prior to the study showed a left-sided canal paresis of 34%.

#### Training progress

During the training process, this patient was able to adapt quickly. Although he did experience slightly increased discomfort after training in the 1^st^ week, he quickly adapted and completed his 2^nd^ and 3^rd^ weeks of training. He was able to perform balance board and tandem stance training. By the end of the 5^th^ week, he was able to complete the exercise at head rotation speeds of 130-140^o^/s. Significant improvements were observed with respect to decreased discomfort and dizziness at work and during daily life activities.

#### Follow-up assessments

One month after the completion of training, the patient was able to complete the DGI and POMA assessments quickly. Although he still exhibited a slight shift in the horizontal head movement assessment, his confidence and activity level had both significantly improved. He still reported feeling slightly dazed at times, but he was generally able to conduct daily activities and perform his regular work duties smoothly.

### Patient 3

#### Brief history and laboratory data from the initial assessments

This patient is a 60-year-old male who worked in accounting and engineering. His work was mostly stationary and required minimal physical activity. He started to experience dizziness after traveling in airplanes prior to the beginning of this study. He noticed a significant body shift while walking and increased dizziness. His dizziness was most severe in extremely light or dark conditions. He found himself becoming increasingly afraid to drive. In particular, he was most anxious about driving his vehicle in heavy traffic or on narrow streets. He found that he was not able to pay immediate attention to traffic when turning around to look over his shoulder and then turning back to the road ahead. As a result, he had to give up most independent travel and came to rely on his family members to provide transportation for him. Because of his symptoms, the patient was depressed, had low self-confidence and was disappointed with his ability to perform his daily activities. His primary goal was to be able to return to safely driving a motor vehicle. The caloric test results 1 week prior to the study showed a left-sided canal paresis of 28%.

#### Training progress

The patient was under a considerable amount of stress, and his training progress was relatively slow. By the time he returned home after the 1^st^ week of training, his family members reported that his body shift while walking had increased. His body shift had significantly improved by the 2^nd^ week of training. At this point, the patient began riding a motorcycle and driving around the neighborhood. He started to experience discomfort and dizziness when checking over his shoulder for oncoming cars. Because of these symptoms, he immediately gave up this initial driving attempt. By the 3^rd^ and 4^th^ weeks of training, he experienced less frequent episodes of severe dizziness. He began to ride his bicycle or motor vehicle on wide streets in his neighborhood. However, he still experienced dizziness when exposed to intense light. By the end of the 6^th^ week of training, the patient was able to drive himself from home (approximately 32.9 kilometers) for his post-test assessment. Between the 5^th^ and 6^th^ weeks of training, he was able to drive himself to work, and he reported increased stress only while driving at high speeds on the highway.

#### Follow-up assessments

One month after training, his overall performance had improved significantly. The SOT and DVA assessments confirmed the persistence of the improvements observed at the completion of training. His DGI and self-confidence assessments also revealed sustained improvements in the training results.

### Patient 4

#### Brief history and laboratory data from the initial assessments

This patient is a 73-year-old woman who had previously worked as a part-time sales representative. One year prior to the start of this study, she started to experience dizziness, which became progressively worse. As a result, she was unable to work. On the day of assessment, she was brought to the clinic in a wheelchair by family members because she was not able to stand or walk for long periods of time. The caloric test showed bilateral vestibular hypofunction. During the initial assessments, she required frequent rest.

#### Training progress

In her first 2 weeks of training, she was only able to perform visual stability training for 3 minutes at a time in a seated position. By the end of the 3^rd^ week, she was able to perform visual stability training with speeds of up to 100^o^/s for 5 minutes at a time. Long periods of rest between trials were still required. During the 3^rd^ and 4^th^ weeks, she was able to perform training in a standing position. A thin cushion was gradually added to interfere with her balance during the training sessions. By the end of training, the patient was able to walk from the parking lot to the training site with only her long-handled umbrella for support. We also observed significant improvements in self-confidence and mood over the course of the training.

#### Follow-up assessments

One month after the completion of training, the patient exhibited significant improvements in both the DGI and POMA assessments. Her degree of body shift was decreased, and she was able to complete the assessments without rest. A slight side-to-side shift was still observed during the DGI assessment, but in general, she was able to complete the assessments without the help of assisting devices. The patient also indicated that she was willing to return to her previous work in sales. She reported large improvements in her ability to perform daily life activities and indicated that her performance had returned to its original state.

## Discussion

The results from this study indicate that 12 sessions of gaze stabilization exercise using interactive video games over the course of 6 weeks can reduce dizziness, improve balance and increase the walking speed of patients with either BVH or UVH, thereby improving their quality of life. These beneficial effects of training can be maintained for at least 1 month after training completion if complemented with in-home training. Because the interactive rehabilitation sessions are fun and challenging, patients can receive more encouragement and confidence through timely feedback. This goal-orientated, task-specific training method has previously been widely used in patients with chronic spinal cord injury, traumatic brain injury, stroke and cerebral palsy [22,40,41].

The major complaints of vestibular patients include imbalance, dizziness, visual confusion or space and motion discomfort (SMD), especially in shopping malls, cars, trains or other places with narrow visual spaces and/or complex and confusing visual stimuli. Virtual reality (VR) technology can provide vestibular dysfunction patients with a controlled environment in which they can gradually acclimate to the scenes that induce their symptoms [16]. Head-mounted displays are easy to carry and affordable, and they were commonly used projection systems for vestibular rehabilitation. However, patients often complain about eye fatigue, blurred vision, headaches, nausea and short-term changes in binocular vision. These adverse effects might stem from changes in the balance between the convergence of the eyeballs [42,43]. Using a head-unrestrained, wide-field gaze shifts the environment; however, no simulator sickness was reported in healthy subjects [16]. Sparto et al. also used a wide-field visual system to evaluate and train patients with vestibular system dysfunction. It was hypothesized that the peripheral flow is an important sensory recalibration for the patients. It was thus concluded that wide-field VR training may be superior to a head mounted-display for the training of vestibular patients [44].

Compared with expensive VR technology, the equipment used in our study is relatively simple and lightweight. It does not require a large amount of space to operate and is therefore suitable for patients’ home training.

During everyday life activities, such as running, the speed of head movement can reach 550°/sec, head acceleration can reach up to 6000°/sec^2^ and the frequency of head rotation can range between 0 and 20 Hz [45,46]. Given this high speed, acceleration and frequency range, it is difficult for patients with loss of vestibular function to adapt, considering the complexity of adaptation and compensation mechanisms, including sensory/motor substitution and central preprogramming [47]. Smooth pursuit, optokinetic function and cervico-ocular reflexes (CORs) can interact with VOR to reduce retinal slip [48]. Smooth pursuit and optokinetic functions are forms of visual compensation for slow head rotation. Animal experiments have confirmed that the neck and vestibular system can interact to generate visual compensation [49]. However, gains in the COR are very slow and can be difficult to observe in primates [50]. Therefore, the clinical significance of these gains has not yet been confirmed. In our study, in addition to gaze stabilization training, the patients turned their heads frequently during the training exercises, which might have led to improvements in the range of head rotation. Whether this might also minimize dizziness by improving neck proprioception requires further study. With regard to the central preprogramming theory, Herdman [51] has proposed that visual acuity is higher during active head movements than during passive head movements in patients with unilateral damage. This finding likely results from the fact that when a patient turns his or her head toward the damaged side, the movement is predictable, and the eyeballs can be adjusted through central preprogramming to maintain gaze stability. In this study, the subjects were asked to perform active head movements. Because the head movements of each subject were self-predictable, compensatory eye movements mediated through central preprogramming were able to improve gaze stability and relieve the patient’s symptoms [52-54]. Discussions on compensatory saccade (CS) have recently gained significant attention. This type of saccade can simultaneously occur during both expected and unexpected head movements. It has been suggested that this compensatory mechanism of the non-vestibular system is related to gaze stability improvements [55,56]. In our study, we did not record the patients’ eye movements; therefore, further investigation is required to determine whether CS has a role in this study.

Our results indicate that the improvement of gaze stability leads to a decreased likelihood of patients experiencing dizziness in response to head movements. The primary functions of the vestibular system are to detect head movement, maintain the stability of images projected on the retinal fovea and maintain postural control during head movements [48]. The vestibule is located in the inner ear, and it functions by detecting the position and movement of the head and by providing the appropriate sensory information to the central nervous system (CNS). Sensory input is primarily sent to the vestibular nuclei for processing and to the cerebellum for the micro-regulation and processing of body balance and coordination. The CNS can then stabilize the head and body via the neural reflexes of the vestibular system. Typically, the CNS maintains the center of gravity of the body between the left and the right foot and takes appropriate actions to maintain body balance. During head movement, the maintenance of body stability relies increasingly on the vestibular system [57]. In our study, the patients were better able to maintain body balance after training. It could be speculated that the vestibulo-spinal reflex was exercised as the training exercises were performed in standing positions. A previous study by Whitney [58,59] has suggested that DGI tends to improve after vestibular rehabilitation in patients with vestibular dysfunction. The results of our study are consistent with that claim. In addition, our data also reveal improved SOT test results as a consequence of the improved balance observed in the study patients.

The computer-aided exercise training system developed in this study was assembled by combining a PC with a simple Bluetooth transmission device. When connected to a Wiimote, this interactive training system can be used immediately after minimal adjustments. The cost of this system is relatively low, and it does not require the installation of any special connection cards. In contrast, commercially available assessment equipment is usually costly. Although other devices (i.e., linear potentiometers and optical gate-switching potentiometers) can be easily acquired, they cannot be easily installed and are not accurate in detecting head movements due to the structural limitations of the human head and neck. The limited angles and movement velocities supported by potentiometers do not meet the requirements for exercise. In addition, most commercially available assessment equipment requires circuit connections, which can result in unnecessary security concerns. In contrast, the light-weight training system developed in our study achieved rapid and practical wireless transmission. The important advantages of this interactive training system include convenience, mobility, safety and low cost.

## Conclusion

Dizziness can lead to serious disability and can significantly affect quality of life. Task-oriented VR interactive training methods provide a new therapeutic approach for the treatment of dizziness. This study established an affordable, easy-to-operate system for task-specific interactive rehabilitation. Our results indicate that stability and gaze stabilization training can improve the recovery of patients with vestibular dysfunction and can help them quickly return to work and daily life activities. This study confirmed the effectiveness of rehabilitation training and could provide a novel therapeutic approach for clinical practitioners. Our results were limited by the observation of only 4 patients; therefore, the recruitment of more dizzy patients with different etiologies would be required to investigate the appropriateness of applying this technique to a wide variety of patients and to determine whether its therapeutic effectiveness is long lasting. This approach may be useful for the establishment of a complete framework for rehabilitation training and for the improvement of the quality of life of vestibular patients.

## Competing interests

The authors declare that they have no competing interests.

## Authors’ contributions

PYC and CLK conceived the study. SHW and PYC designed, developed and integrated the training system. WLH and CLK performed the evaluation and training. PYC and CLK drafted the manuscript and revised it critically. All authors read and approved the final manuscript.
